# Personalized medicine using omics approaches in acute respiratory distress syndrome to identify biological phenotypes

**DOI:** 10.1186/s12931-022-02233-0

**Published:** 2022-11-19

**Authors:** Denise Battaglini, Lou’i Al-Husinat, Ana Gabriela Normando, Adriana Paes Leme, Kleber Franchini, Marcelo Morales, Paolo Pelosi, Patricia RM Rocco

**Affiliations:** 1 Anesthesia and Intensive Care, San Martino Policlinico Hospital, Instituto di Ricovero e Cura a Carattere Scientifico (IRCCS) for Oncology and Neuroscience, Genoa, Italy; 2 Department of Surgical Science and Integrated Diagnostics (DISC), University of Genoa, Genoa, Italy; 3 Department of Medicine, University of Barcelona, Barcelona, Spain; 4 Department of Clinical Medical Sciences, Faculty of Medicine, Yarmouk University, P.O. Box 566, Irbid, 21163 Jordan; 5 Brazilian Biosciences National Laboratory, LNBio, Brazilian Center for Research in Energy and Materials, CNPEM, Campinas, Brazil; 6 Laboratory of Cellular and Molecular Physiology, Carlos Chagas Filho Biophysics Institute, Federal University of Rio de Janeiro, Rio de Janeiro, Brazil; 7 Laboratory of Pulmonary Investigation, Carlos Chagas Filho Biophysics Institute, Federal University of Rio de Janeiro, Rio de Janeiro, Brazil

**Keywords:** Omics, Proteomics, Genomics, Transcriptomics, Metabolomics, ARDS, Genotypes

## Abstract

In the last decade, research on acute respiratory distress syndrome (ARDS) has made considerable progress. However, ARDS remains a leading cause of mortality in the intensive care unit. ARDS presents distinct subphenotypes with different clinical and biological features. The pathophysiologic mechanisms of ARDS may contribute to the biological variability and partially explain why some pharmacologic therapies for ARDS have failed to improve patient outcomes. Therefore, identifying ARDS variability and heterogeneity might be a key strategy for finding effective treatments. Research involving studies on biomarkers and genomic, metabolomic, and proteomic technologies is increasing. These new approaches, which are dedicated to the identification and quantitative analysis of components from biological matrixes, may help differentiate between different types of damage and predict clinical outcome and risk. Omics technologies offer a new opportunity for the development of diagnostic tools and personalized therapy in ARDS. This narrative review assesses recent evidence regarding genomics, proteomics, and metabolomics in ARDS research.

## Background

In the latest decade, research on acute respiratory distress syndrome (ARDS) has made considerable progress in understanding the pathophysiology of the disease, diagnostic criteria, biomarkers, and rescue therapies, but it remains a leading cause of mortality in the intensive care unit (ICU) [1]. Current therapies for ARDS are mainly supportive. The failure of pharmacologic therapies for ARDS has been explained by the clinical, pathophysiologic, and biological heterogeneity of this syndrome [2]. Research on the study of biomarkers and genomic, metabolomic, and proteomic technologies is increasing. These novel approaches, which are dedicated to the identification and quantitative analysis of components from biological matrixes, may help to differentiate between different types of damage and predict clinical outcome and risk [3, 4]. Omics technologies offer a new opportunity for the development of diagnostic tools and personalized therapy in ARDS [5]. Thus, this narrative review compiles the most recent findings regarding genomics, proteomics, transcriptomics, and metabolomics approaches in ARDS research.

### Current therapies for ARDS

Increasing effort has been made to elucidate which treatments or supportive interventions can be used [6, 7]. Existing treatments for ARDS are mainly supportive [8]. Using the general definition of ARDS based on the Berlin criteria, randomized controlled trials (RCTs) have found some supportive treatment strategies that can be generalized for all patients with ARDS. In contrast, pharmacologic treatments and some possible supportive therapies may benefit from personalization; specific physiologic thresholds, clinical characteristics, biological or omics subphenotypes have been targeted to find treatable traits. Supportive treatments for ARDS include protective mechanical ventilation using a low tidal volume (4–6 mL/kg of predicted body weight), plateau pressure (< 28–30 cmH_2_O) [9, 10], low driving pressure (< 13–15 cmH_2_O), and individualized levels of positive end-expiratory pressure (PEEP) [7]. In the case of refractory hypoxemia, neuromuscular blocking agents, prone positioning, recruitment maneuvers, extracorporeal membrane oxygenation should be considered [7]. Several drugs that have been tested over the years failed to demonstrate potential efficacy [8]. Current therapies include neuromuscular blocking agents, sedatives, and analgesics. RCTs that have investigated pharmacologic interventions in ARDS have not shown consistent beneficial treatments with high potential for failed drug discovery [11]. Failure of clinical trials in ARDS can be attributed to the fact that the heterogeneity of this syndrome may have affected the results. Pharmacotherapies usually do not target a specific subpopulation of patients with ARDS. Trials design should account for proper selection of patients based on their biological and clinical characteristics. In this context, omics approaches may help to identify the correct subphenotypes of patients with ARDS who can benefit from a specific pharmacotherapy [8].

### ARDS classification and phenotyping

ARDS is a syndrome that can be caused by various diseases. Over the years, experimental and clinical research has focused on identifying the causative factors of ARDS heterogeneity [2]. The increased interest in addressing ARDS heterogeneity led to several clinical studies that tried to identify subphenotypes of patients with ARDS according to clinical features (i.e., dead space fraction, PEEP, ventilatory ratio, driving pressure) or biological features (i.e., specific inflammatory and coagulative biomarkers) [12–16], the causes of ARDS (i.e., pulmonary vs. extrapulmonary, acute kidney injury vs. not, trauma vs. non-trauma) and time of ARDS diagnosis (before vs. 48 h after ICU admission) [17–19], as well as stratification by omics into genotypes, i.e., the genetic material that contributes to phenotypes [20]. According to ARDS subphenotypes, we define an endotype as a subtype of a disease condition that is characterized by a distinct pathophysiologic mechanism. ARDS subphenotypes may be associated with outcome and stratify patients at the bedside, thus selecting patients according to different therapeutic strategies. However, several concerns have been identified when ARDS was classified according to the different subphenotypes: (1) broad variation in the recruited population, (2) distinct and variable timing for the assessment of biomarkers, and (3) poor association between physiologic changes and validation of biomarkers [21].

### Clinical classification of ARDS

Risk stratification of patients with ARDS started in 1967 when ARDS was described as a form of hypoxemic respiratory failure due to non-cardiogenic pulmonary edema with increased work of breathing and reduced compliance of the lungs [22]. In 1992, the American-European Consensus Conference developed the first consensus to define ARDS [23]. In 2012, another consensus conference in Berlin defined ARDS as a syndrome with an acute onset within 7 days of insult, and risk stratification was suggested by categorizing patients as mild, moderate, or severe according to the ratio of arterial oxygen tension (PaO_2_) to fraction of inspired oxygen (FiO_2_) (with PEEP of 5 cmH_2_O or more) at ARDS onset [24]. On the clinical side, ARDS can be classified as pulmonary or extrapulmonary, depending on the pathogenic pathway [25]. When a direct insult to the alveolar epithelium causes a local alveolar inflammatory response, ARDS is defined as pulmonary; an indirect insult that affects the vascular endothelium through the bloodstream causing inflammation is defined as extrapulmonary ARDS [26].

### Histopathologic classification of ARDS

Diffuse alveolar damage (DAD) is considered to be the typical histologic pattern of ARDS, but only half of patients exhibit this morphologic hallmark [27]. Interstitial and alveolar edema, hyaline membrane, alveolar hemorrhage, neutrophil infiltration, fibrin deposition, and atelectasis are features of DAD; the latter may evolve into a fibroproliferative stage and fibrotic disease [22].

### Radiologic classification of ARDS

Radiologic studies revealed different lung patterns (i.e., focal [lung areas of attenuation predominating in the lower lobes or gravitationally dependent parenchyma] or diffuse [lung areas of attenuation distributed diffusely across the lungs]) among patients with ARDS. A diffuse radiologic pattern is associated with worse outcome [28–30]. According to radiologic subphenotypes, the CESAR trial adopted a Murray Lung Score > 3 points in patients with ARDS under extracorporeal membrane oxygenation (ECMO) [31]. Similarly, the RALE score, which includes a radiologic evaluation of patients with ARDS, was associated with 28-day mortality [32]. A recent study (LIVE trial) compared a personalized mechanical ventilation strategy, selected according to radiologic subphenotypes, with a standard lung protective ventilation strategy and found better outcomes with the personalized strategy [33]. No difference in 90-day mortality was found between the personalized and control groups. When patients were reallocated according to the focal or non-focal nature of ARDS, a significant difference in mortality was found between the groups [33].

### Biological phenotypes of ARDS

ARDS presents substantial heterogeneity with regard to biological biomarkers. Using stepwise modeling of latent class analysis to find phenotypes based on clinical data and plasma biomarkers, Famous et al. [34] confirmed the existence of 2 subphenotypes: one characterized by hyperinflammation and hypotension, and the other characterized by a hypoinflammatory status. These 2 subphenotypes demonstrated accuracy to identify which subpopulation of patients with ARDS can benefit from a conservative or liberal fluid strategy. This confirmed the existence of different subphenotypes among patients with ARDS, as reported in previous re-analysis of RCTs by Calfee et al. [35] and Sinha et al. [36]. Calfee et al. [35], using a latent class analysis with 8 plasma biomarkers [37], distinguished between a “hyperinflammatory” and a “hypoinflammatory” phenotype in patients with ARDS, whereas Bos et al. [15] identified an “uninflamed” and a “reactive” phenotype. However, inflammatory biomarkers are usually unspecific and may not be characteristic in ARDS [38]. In this context, biological biomarkers associated with endothelial damage (i.e., angiopoietin-2, intracellular adhesion molecule-1), epithelial cell damage (i.e., soluble receptor for advanced glycation and products, surfactant protein-D), inflammation (i.e., interleukin [IL], tumor necrosis factor-α [TNF-α]), and coagulation (i.e., protein C, plasminogen activator inhibitor-1, fibrinogen, D-dimer) have been described [39, 40] and associated with different subphenotypes of ARDS, which may partially explain why some pharmacologic therapies for ARDS have failed to improve patient outcomes [41]. Numerous genomic, proteomic, transcriptomic, and metabolomic markers have been studied to find subphenotypes of patients with ARDS who share important biological features with an impact on clinical outcome [39]. Several studies have confirmed the association between ARDS subphenotypes and different treatment responses or outcomes [34, 42]. Understanding the importance of ARDS subphenotypes and their impact on patient outcome is important to plan and conduct new research projects evaluating specific therapies.

## Omics in ARDS research

**Fig. 1:**
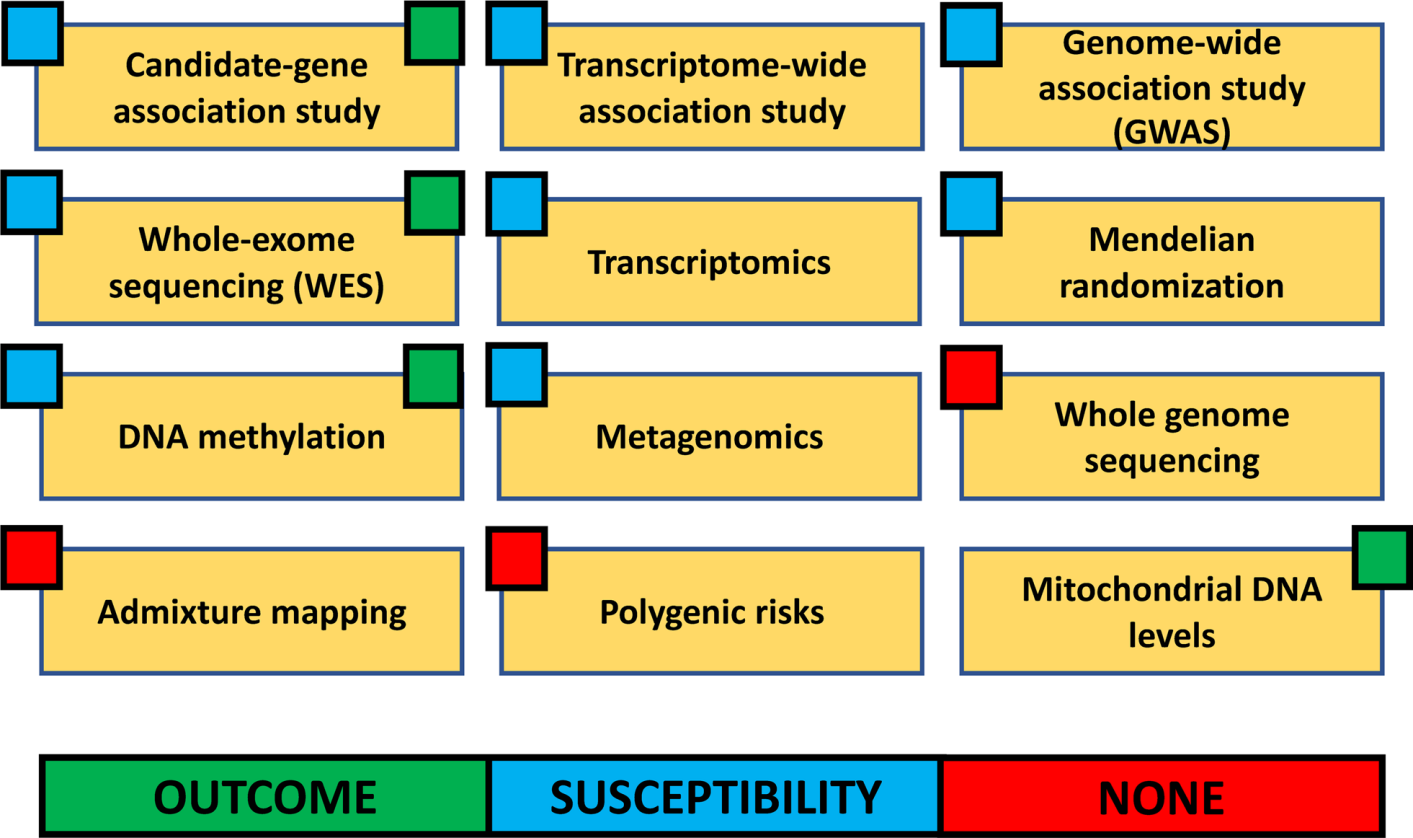
Main omics approaches applicable to ARDS (outcome, susceptibility, none)

The identification of new disease-specific biomarkers is a leading approach to current research design and goals for ARDS. With the lack of effective pharmacologic therapy and high disability and mortality, advances in ARDS research have been focusing on promising technologies such as genomics, transcriptomics, proteomics, and metabolomics. Genomics refers to the ensemble of genes; transcriptomics refers to the study of ribonucleic acid molecules within a sample, providing a link between genomics and proteomics; proteomics refers to the proteins translated in an organism; and metabolomics refers to the small molecules (metabolites) identified within a biological sample [43]. Therefore, we conducted a systematic search on 4 databases (PubMed, EMBASE, Scopus, and Cochrane) up to 1 September 2022 to identify studies regarding omics approaches in ARDS research and clinical implications to present make this narrative review of the literature as comprehensive as possible. The main omics approaches applicable to ARDS and the phenotypes assessed (outcome, susceptibility, none) are reported in Fig. 1.

### Genomics

**Fig. 2:**
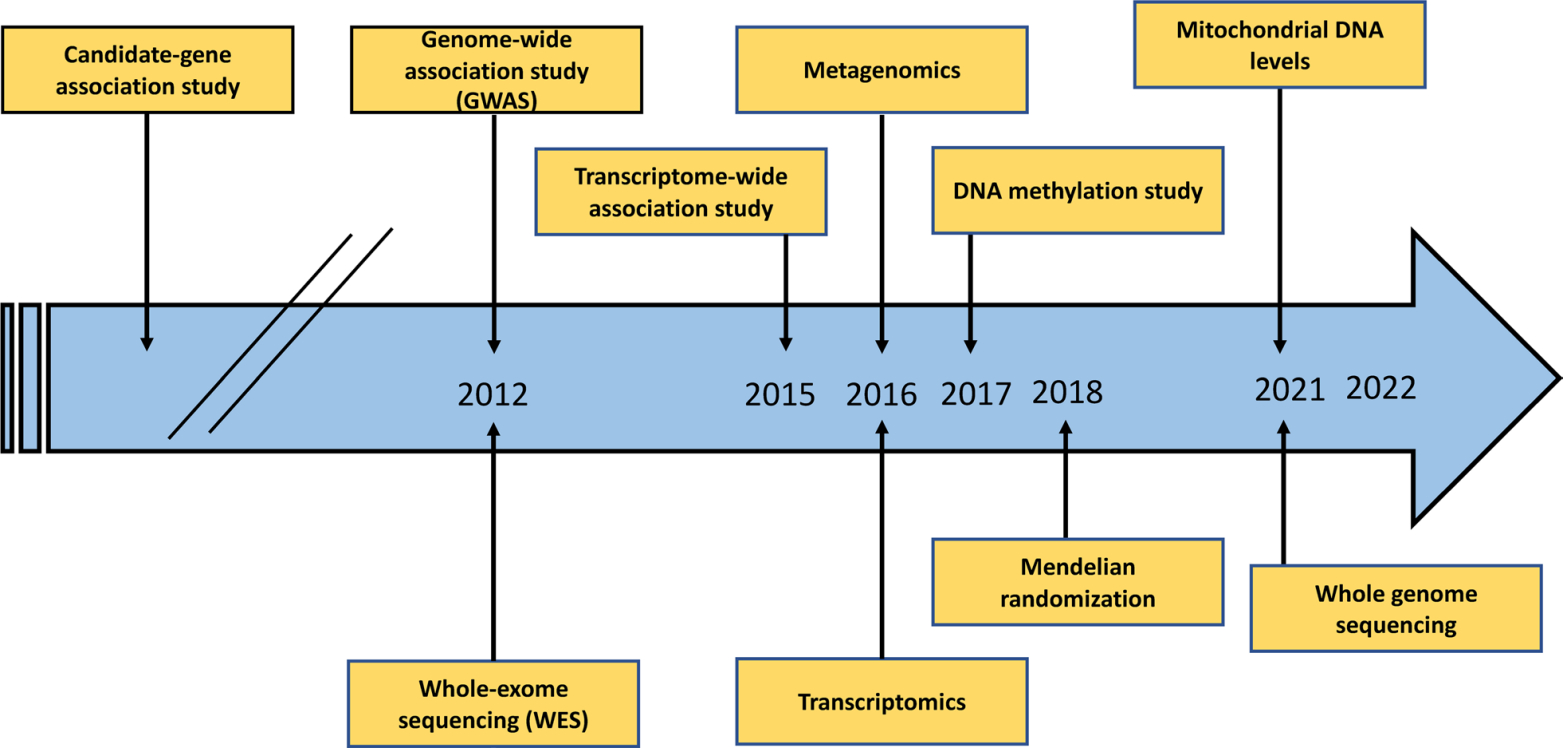
Update on genomic research in ARDS up to 2022. Modified from Hernandez-Beeftnik T, Guillen-Guio B, Villar J, Flores C. Genomics and the acute respiratory distress syndrome: current and future directions. Mol Sci. 2019;20(16):4004

ARDS is a complex disease that activates various biological patterns that can be detected using biomarkers of lung injury [44]. Genomics is the study of genes and genetic variants of a condition, including interactions of genes with each other and with the environment [45]. Genomics has led to advances in knowledge of human disease, identifying novel pathways and genetic variants associated with human pathologic conditions. The objective of genomic technologies is to identify ARDS hyperinflammatory subphenotypes with higher risk for death or susceptibility. Many genes have limited value for risk prediction, although their aggregated impact on lung injury phenotypes in ARDS pathology is interesting and promising [44]. Since 2000, the genes that are associated with ARDS have been identified through different approaches, including a candidate gene approach [46–49], micro-array analysis [47, 50–52], whole-genome genotyping [53], and whole-exome sequencing [54, 55]. The first candidate gene study on ARDS dates to 1992 (on angiotensin-converting enzyme [*ACE*] polymorphism) [56], the first candidate gene study validation was developed in 2000 and 2002 [57, 58], the first gene and genome-wide association study (GWAS) was developed in 2012 [53], and the first next-generation sequencing dates to 2014 [54]. Since then, genomic research has made progress, targeting the cellular and molecular mechanisms of ARDS. Particularly, genomic research in ARDS has focused on the identification of genes that might be modulated for prevention and treatment of ARDS, targeting alveolar-capillary barrier dysfunction, alveolar fluid clearance dysfunction, and systemic inflammation [59]. Shortt et al. [54] identified a novel single nucleotide polymorphism (i.e., the presence of genetic variation within a population) associated with ARDS by exome sequencing as a potential novel biomarker in ARDS research. GWAS is the current genetic approach used in ARDS research. The evolution of genomic research over time is presented in Fig. 2.

The first GWAS study was developed with the aim of identifying risk variants for ARDS. This study identified the gene PTPRF interacting protein alpha 1 [*PPFIA1*] as a potential functional candidate for future research on ARDS in major trauma [53]. GWAS technology may help to predict ARDS risk and susceptibility. GWAS genetic variants were tested preferentially in white people, and only one study reported GWAS on African Americans [60]. A large GWAS was conducted on both Europeans and African Americans and reported that a novel locus within the gene BLOC-1 related complex subunit-5 [*BORCS5*] was a predictor of ARDS susceptibility in Europeans [61]. One of the genes more strongly associated with mortality in ARDS was the FER gene called rs4957796, which was strongly associated with 28-day survival in patients with sepsis and pneumonia [62]. Associations between FER genetic variants and mortality in ARDS have been confirmed by further studies [62, 63]. The main genes identified for the prediction of susceptibility and outcome in ARDS according to the pathophysiologic mechanism of ARDS are presented in Table 1 [45, 57, 58, 60–101].

**Table 1:** The main genes identified for the prediction of susceptibility and outcome in ARDS according to the pathophysiologic mechanism

Mechanism of ARDS	Gene
Vascular permeability [60, 61, 64–74, 99–101]	MAP3K1, FLT1, ANGPT2, AGT, EPAS1, HSPG2, KLK2, MAP3K1, MAP3K6, MYLK, NAMPT, SELPLG, S1PR3, VEGFA
Immune response [57, 75–88]]	IL17, DEFB1, FER, AGER, sRAGE, CHIT1, FKB1, IL1B, IL1RN, IL4, IL6, IL10, IL13, IL17, IL18, IL32, IRAK3, LTA, MBL2, MIF, NFKBIA, PDE48, PI3, SELPLG, SFTPA1, SFTPA2, SFTPD, STAT1, TIRAP, TLR1, TNF, TNFRSF11A, TRAF6
Oxidative stress [89–93]	EGLN1, FTL, HIF, HIF2a, HMOX1, HMOX2, NFE2L2, NQO1, SOD3
Epithelial injury [58]	SP-A, SP-B
Apoptosis	FAS
Chemotaxis	CXCL2, CXCR2, DARC, ISG15
Fibrosis [94]	HAS1, MUC5B, SERPINE1
Cell growth [95–97]	AQP5, ADGRV1, BCL11A, EGF, FZD2, GHR, POPDC3, TGFB2
Coagulation [98]	F5, GP5, LRRC16A, PLAU, VWF
Metabolism [45]	ADA, ADIPOQ, AHR, APOA1, CBS, CYP1A1, DIO2, FAAH, PPARGC1A, PRKAG2, UGT2B7, VLDLR
Other mechanisms [45]	ABCC1, ADRBK2, CLASRP, GADD45A, GRM3, HTR2A

*ACE* angiotensin-converting enzyme, *ADMR2* adrenomeddulin-2, *AGER* advanced glycosylation end-product specific receptor, *ANGPT2* angiopoietin-2, *AQP1* acquaporin-1, *ARL3* ADP ribosylation factor like GTPase, *ARSD* arylsulfatase-D, *BTG1* antiproliferation factor-1, *CCL2* chemokines ligand-2, *CD* cluster differentiation, *CEBPA* CCAAT enhancer binding protein alpha, *CLEC4E* C-type lectin like domain 4e, *COX* cyclooxygenase, *CREBPZ* pancreatic beta cell-specific gene, *CXCR* chemokine receptor, *DEFB1* defensin beta-1, *DIO1* iodothyronine deiodinase-1, *EGF* epidermal growth factor, *EGLN* prolyl hydroxylase encoding gene, *F3* tissue factor precursor, *FAAH* fatty acid amide hydrolase, *FAS* Fas cell surface death receptor, *FER* FER tyrosine kinase, *FGA* fibrinogen A, *FTH* ferritin heavy chain, *FTL* ferritin light chain, *GADD45A* growth arrest and DNA damage-inducible-45, *GJA* gap junction alpha, *HCAR* constitutive androstane receptor, *HMOX* heme oxygenase-1, *IL* interleukin, *IRAK* interleukin-1 receptor associated kinases, *ITGB1* integrin beta-1, *KXR* X-linked Kx, *LCN2* lipocain-2, *LRRC* leucine-rich repeat containing, *LTA* lymphotoxin alpha, *MAP* mitogen-activated protein kinase-3, *MBL* mannose-binding lectin, *MIF* macrophage migration inhibitory factor, *MME* membrane metalloendopeptidase, *MUC5B* mucin5B, *MYLK* myosin light chain kinase, *NAMPT* nicotinamide phosphoribosyl transferase, *NFKB* nuclear factor kappa-B, *NPEPL* aminopeptidase-like, *NQO1* NADPH dehydrogenase (quinone), *NRF2* nuclear factor erythroid-2 related factor 2, *OLFM* olfactomedin-1, *PI3* peptidase inhibitor-3, *PLAUR* urokinase plasminogen activator receptor, *PNPLA* patatin-like phospholipase domain-containing protein-3, *POPDC3* Popeye domain-containing protein-3, *PPFIA* protein tyrosine phosphatase, *RBP7* retinol binding protein-7, *SERPINE* serine protease inhibitor-1, *SFTPB* surfactant protein-B, *SOC* suppressor of cytokine signaling, *SOD3* superoxide dismutase, *TEK* tyrosine kinase receptor, *TFF* trefoil factor family, *THBS1* thrombospondin-1, *TIRAP* TIR domain-containing adaptor protein, *TLR* toll-like receptor, *TNF* tumor necrosis factor, *VEGF* vascular endothelial growth factor

Although modulation of genes could alleviate certain symptoms of ARDS, a single gene or combination of genes responsible for ARDS has not been identified yet in experimental animal research or in human studies [59]. Genomic research has great potential to elucidate ARDS pathways by identifying genetic associations and biomarkers, but ARDS is a challenging condition that may limit genomic research for various reasons: (1) ARDS is a syndrome that is a consequence of other pathologic conditions such as sepsis, trauma, or pneumonia, (2) ARDS is a syndrome that is a consequence of other pathologic conditions such as sepsis, trauma, or pneumonia, (3) ARDS lacks specific diagnostic tests and is often underrecognized, (4) blood samples for genomic research in ARDS may not reflect the expression pattern of lung endothelium or epithelium because gene expression is tissue specific, and only 10% of patients with ARDS undergo lung biopsy [102]; and (5) the epigenetic influence on disease susceptibility and outcome. This latter point is of particular interest because epigenetic changes in ARDS, probably induced by environmental interactions such as mechanical ventilation or infection, may contribute to modifications in gene expression, function, or activity without changing deoxyribonucleic sequences [103]. Genomics approaches need to be implemented in daily clinical practice to allow better understanding of ARDS and therapies and to design new clinical trials, offering a possible re-assessment of certain drugs that failed to provide benefits when administered indiscriminately to all patients with ARDS [104]. The COVID-19 pandemic has been a unique challenge and the global effort during the pandemic has led to and reinforced collaborations. This incredible effort to find effective therapeutics, also including genomics solutions, should be reconsidered within the context of ARDS research [105].

### Transcriptomics

The cellular process of transcription produces ribonucleic acids (RNAs) that are based on the genomic template. The human genome is composed of approximately 21,000 protein-coding genes and several noncoding RNA genes [106]. The proteins are assembled through the process of transcription whereby RNAs are processed and spliced into mature forms. The messenger RNA (mRNA) transcripts and codes regions that promote the translation of proteins. Further, transfer RNA (tRNA), ribosomal RNA (rRNA), small nuclear RNA (snRNA), small nucleolar RNA (snoRNA), short interfering RNA (siRNAs), micro-RNA (miRNAs), long noncoding RNA, and pseudogenes are involved in several cellular activities. Different from DNA-based analysis, transcriptomics allows the study of the passage of information through cell lineages, using RNA both as carrier and catalytic [107]. RNAs have tissue-specific patterns of expression that are specific and time dependent. With post-transcriptional regulation and degradation, RNA and protein expression are not always correlated. Therefore, the transcriptome includes RNAs, protein-coding, non-protein-coding, alternatively spliced, polyadenylate, initiated, sense, antisense, and RNA-edited transcripts.

The advantage of transcriptomics is that the genome-wide investigation offers a global picture instead of giving excessive importance to a single candidate gene [107]. We provide a brief overview of the current advances in transcriptomics for ARDS, specifically focusing on the most investigated techniques (miRNA and mRNA).

#### miRNA

Much of the evidence regarding miRNA identification in ARDS comes from preclinical studies. MiRNAs are involved in several pathophysiologic processes, including regulation of cell proliferation, differentiation, apoptosis, metabolism, and the immune response [108]. Some transcriptomics analyses of miRNAs in preclinical models revealed that inflammation during ARDS promotes macrophage proliferation and inflammation, also of the lung [109–112]. With regard to clinical evidence in humans, some miRNAs have been proposed as biomarkers of the pathophysiology, risk, and mortality in ARDS [113, 114]. Blood leukocytes of patients with ARDS showed increased expression of miRNA, and steroid therapy had no effect on the miRNA identified in patients with ARDS [115]. The clinical significance of miRNA up-/downregulation [116–127] in humans is presented in Table 2.

**Table 2:** Clinical significance of miRNA up-/downregulation in humans with ARDS

References	Micro-RNA	Model	Effect	Clinical significance
Lee et al. [123]	miR-106a-5p, miR-17-5p, miR-29a-3	All-cause ARDS	Upregulated	Prediction of mortality
	miR-126-3p, miR-191-5p, miR-223-3p		Downregulated	
Wang et al. [116]	miR-103, miR-107	Sepsis-associated ARDS	Downregulated	Increased risk of ARDS and 28-day mortality
Xu et al. [117]	miR-92	Sepsis-associated ARDS	Upregulated	Risk of ARDS
Wang et al. [120]	miR155	Sepsis-associated ARDS	Upregulated	Association with inflammatory lung injury
Wu et al. [121]	miR-27a, miR-126, miR-146a, miR-155	Pneumonia-associated ALI	Upregulated	Risk of ARDS
Rahmel et al. [124]	miR-122	All-cause ARDS	Upregulated	Association with acute liver injury and ARDS
Li et al. [125]	miR-140	ALI except sepsis	Downregulated	Association with ALI and inflammation
Zhu et al. [126]	miR-181a, miR-92a	All-cause ARDS	Upregulated	Risk of ARDS
	miR-424		Downregulated	
Goodwin et al. [122]	miR-887-3p	Sepsis-associated ARDS	Upregulated	Risk of ARDS
Lu et al. [127]	miR-22-3p, 1260b, 762, 23b, 23a	AP-associated ARDS	Upregulation	Risk of pancreatitis and ARDS
	has-miR-550a, 324-5p, 484, 331-3p, 22-3p, 140-3p, 342-3p		Downregulated	
Shi et al. [118]	miR-127	AP-associated ALI	Upregulated	Association with acute pancreatitis and ARDS
	miR-199a	Sepsis-associated ARDS	Downregulated	Risk of ARDS
Zhu et al. [119]	miR-628-3p, miR-766, miR-922, miR-7, miR-194	All-cause ARDS	Upregulated	Increased 28-day mortality

*miR* micro-RNA, *ARDS* acute respiratory distress syndrome, *AP* acute pancreatitis

Therapeutic strategies for ARDS by modulating the transcription of genes have been proposed in the preclinical setting. Through regulation of the miR-423-5p/FOXA1 axis, silencing long noncoding RNA H19 alleviates pulmonary injury, inflammation, and fibrosis in a rat model of ARDS [128]. In rats, metformin relieved ARDS by reducing miR-138 expression [129], and mesenchymal stromal cells modulated the response in an experimental sepsis-ARDS model in rats by regulating the expression of miR-27a-5p [130]. However, the application of these therapies in the clinical setting is still limited. A limited number of studies on miRNAs have been published to date, although they found a clear association with the occurrence of inflammation in ARDS by affecting macrophages and other inflammatory pathways [131].

#### mRNA

Preclinical studies showed interesting results with mRNA technology in ARDS models. A study in mice identified that increased mRNA and protein expression of ELAVL-1/HuR and GSK3β was associated with difficult resolution of ARDS [132]. In another experimental murine model, heme oxygenase-1 mRNA and protein expression were increased in mice that developed malaria-associated ARDS [133]. In another mouse model of ARDS, several mRNAs were hypo-expressed, including METTL16, FTO, METTL3, KIAA1429, RBM15, ALKBH5, YTHDF2, YTHDF3, YTHDC2, and IGFBP2, and were associated with m6A methylation caused by administration of lipopolysaccharide, which was involved in the regulation of inflammation and the development of lung injury [134]. From these preclinical models, it seems clear that mRNA might be modulated to reduce inflammation in ARDS. Some therapeutic agents have also been investigated for modulating mRNA in experimental models of ARDS. Photobiomodulation of human mesenchymal stromal cells, and suppression of incRNA HOTAIR can interfere with the inflammatory response [135–137]. Andrographolide sulfonate treatment improves alveolar hypercoagulation and fibrinolytic inhibition and attenuates lung inflammation in an ARDS model in mice by inactivating the nuclear factor-κB pathway [138].

However, clinical evidence on mRNA technology in ARDS is still limited, although the results are interesting. The mRNA profile of patients with ARDS has been investigated by Ning et al. [139] who found 242 and 102 differently expressed genes in GSE32707 and GSE66890, respectively. Inflammasome-related mRNA transcripts (CASP1, IL1B, and IL18) were increased in peripheral blood of patients with sepsis- or trauma-induced ARDS [140]. The mRNA encoding proinflammatory cytokines MyD88 and IRAK1 in mononuclear cells of peripheral blood have been investigated in patients with ARDS and healthy controls; no proinflammatory alterations were found in patients with ARDS [141]. Comparing mRNA in 300 patients with ARDS and 300 controls, TNF-α rs1800629, IL-6 rs1800796, and MyD88 rs7744 SNPs were identified as markers of increased risk for ARDS and a poor prognosis [142]. N-Methyl-adenosine modification of Trim59 RNA was protective against the risk of developing ARDS during sepsis [143]. The mRNA levels of p300, CREB binding protein, tyrosine-protein kinase transmembrane receptor γt, and plasma concentration of IL-17, IL-6 were higher in patients with acute ARDS compared with controls, whereas p300/CBP expression was a risk factor for 28-day mortality [144]. All these studies, although at a preliminary stage of transcriptomics research in ARDS, demonstrate that investigations are advancing rapidly and could be a valuable support to identify biological phenotypes, thus helping to better understand the pathophysiology of ARDS and provide potential new targets for the development of effective RCTs on a specific subphenotype of patients with ARDS.

### Proteomics

Proteomics is the analysis of the proteins translated in cells, tissues, and organisms at a specific time to investigate physiologic and disease conditions, molecular mechanisms, and diagnostic and prognostic biomarkers [145]. Proteomics can also identify and quantify post-translational protein modifications, localizations, activities and functions, and protein–protein interactions [146, 147]. Over the years, several proteomic technologies, together with advances in instrumentation, have been developed (e.g., shotgun two-dimensional high-performance liquid chromatography tandem mass spectrometry [148], matrix-assisted laser desorption/ionization time of flight mass spectrometry [149], and others) and have helped to quantify many proteins that were not detected with traditional methods. Proteomic research can be either targeted or untargeted, depending on multiple or single analysis of known proteins with potential for investigating disease progression or identifying biomarkers. Untargeted proteomics, also known as discovery proteomics, can be adopted to identify several proteins associated with a disease, detect several features in a single analysis, identify potential related biomarkers, and may allow large-scale studies. Despite these advantages, discovery proteomics has limit ability to quantify proteins compared with targeted proteomics, which provides higher sensitivity and accuracy for quantification of a predefined set of targeted proteins usually selected from previous discovery proteomics [150]. The advantages of targeted proteomics include the ability to select candidates to investigate their abundance in subtypes of a disease, higher analytical precision, although with a possible risk of limiting the response of interest, and limited knowledge of the protein of interest, resources, and sample size [146]. Proteome analysis in patients with ARDS can be run on various tissues and cell types, including plasma, lung tissue, lung cells, and bronchoalveolar lavage fluid (BALF) [43]. A list of proteomic studies in ARDS according to the sampling process is provided in Table 3.

**Table 3:** Update on proteomic studies in ARDS

Type of sample	References	Study aims	Number of patients	Proteomics analysis	Conclusions
Plasma	Chen et al. 2013 [151]	To examine the changes in serum protein expression in patients with ARDS	11 patients and 15 controls	iTRAQ, MALDI-TOF, LC–MS/MS	Of the 132 proteins identified, 16 were expressed in patients with ARDS, of which 11 overlapped between the direct and indirect lung injury groups; 5 were specific to the indirect lung injury group
	Liu et al. 2017 [152]	To examine the changes in serum protein expression in patients with ARDS undergoing ECMO	51 patients	FC, ELISA, MS	The levels of IL10 were correlated with survival and delayed recovery
	Li et al. 2019 [153]	To examine the changes in serum protein expression in patients with ARDS	5 patients and 5 healthy controls	LC–MS/MS	Of the 162 proteins identified, 128 were upregulated and 34 were downregulated in patients with ARDS
	Dong et al. 2021 [154]	To examine the changes in serum protein expression in patients with ARDS and association with 28-day mortality	300 patients	SomaScan assay	IGFBP7 moderately increased ARDS 28-day mortality. The association between IGFBP7 and ARDS 28-day mortality seems to be mediated by the platelet count
BALF	Bowler et al. 2004 [155]	To examine the protein profile of patients with mild ARDS and healthy volunteers	16 patients and 12 healthy volunteers	2D-PAGE, MALDI-TOF/MS	Of the 158 proteins identified, transferrin, IgG, clusterin, serum amyloid protein, hemopexin, IgG heavy chain, complement component 3, α2 or β‑hemoglobin, α2 or β2‑glycoprotein1, and α2‑Heremans‑Schmid‑glycoprotein were upregulated in patients with ARDS; SP-A and α1 antitrypsin were downregulated
	de Torre et al. 2006[156]	To investigate the temporal changes of proteome in patients with ARDS and healthy volunteers	11 patients and 33 healthy volunteers	2D-PAGE, MALDI-TOF, SELDI-TOF	Apolipoprotein A1, S100 calcium-binding proteinsA8 and A9, AT3, transthyretin, hemoglobin-A chain-b were upregulated in ARDS
	Chang et al. 2008 [149]	To examine the changes in protein expression in patients with ARDS over time	20 patients and 9 healthy volunteers	2D-PAGE, MALDI-TOF/MS	991 proteins were detected, of which those implicated in the immune response (complement C3, S100 calgranulin A9, fibrinogen alpha chain, α1 antitrypsin, apolipoprotein A1, and hemopexin) were overexpressed
	Schnapp et al. 2013[148]	To examine the changes in protein expression in patients with ARDS	3 patients	2D-HPLC, LC–MS/MS, Shotgun	870 proteins were identified; albumin, ceruplasmin, fibrinogen α chain, α1 chymotrypsin, α2-HS-glycoprotein, antitrypsin inhibitor, IGFBP-3 were upregulated
	Nguyen et al. 2013[157]	To examine changes in proteins in patients with ARDS with and without VAP	30 patients	2D-HPLC, ESI–MS/MS	In patients with ARDS, 119 proteins were identified, of which 47 were downregulated; S100A8, lactotransferrin, and actinin 1 were upregulated in VAP-positive patients
	Bhargava et al. 2014[158]	To examine the changes in protein expression in early-phase survivors vs non-survivors and late phase survivors with ARDS	24 patients	iTRAQ, 2D-HPLC, LC–MS/MS	Patients with ARDS showed predominance in coagulation and fibrinolysis proteins, immune responsive proteins, and proteins maintaining cation and iron homeostasis. On the other hand, early-phase non-survivors had more proteins of the carbohydrate catabolism
	Ren et al. 2016 [159]	To examine changes in proteins in patients with ARDS and pneumonia	14 patients	iTRAQ, LC–MS/MS, 2D-HPLC, LC–MS/MS	DMBT1 can potentially serve as a biomarker for early ARDS diagnosis and disease severity assessment
	Bhargava et al. 2017 [160]	To examine changes in proteins in patients with ARDS	36 patients	iTRAQ, 2D-HPLC, LC–MS/MS	1115 high confidence proteins in BALF were identified, of which 142 were differentially expressed between survivors and non-survivors
Lung cell	Dong et al. 2013 [161]	To examine changes in proteins in alveolar macrophages of patients with ARDS with and without VAP	14 patients	2D-PAGE, MALDI-TOF/MS	Between alveolar macrophages, 135 proteins were differentially expressed, of which 17 were characteristic of the recovery phase, and 10 of the exudative phase

*ARDS* acute respiratory distress syndrome, *AT* antithrombin, *BALF* bronchoalveolar lavage fluid, *DMBT1* deleted in malignant brain tumors 1 protein, *ECMO* extracorporeal membrane oxygenation, *ESI* electrospray ionization, *IGFBP* plasma insulin-like growth factor binding protein, *IL* interleukin, *VAP* ventilator-associated pneumonia, *iTRAQ* isobaric tags for relative and absolute quantitation, *HPLC* high-performance liquid chromatography, *MS* mass spectrometry, *LC* liquid chromatography, *MALDI-TOF* matrix-assisted laser desorption ionization time of flight, *FC* flow cytometry, *ELISA* enzyme-linked immunosorbent assay, *SomaScan assay* Slow Off-Rate Modified Aptamers Scan assay, *SELDI-TOF* surface-enhanced laser desorption/ionization time of flight

#### Plasma proteome

Human plasma has great potential for the identification of proteins that may have diagnostic and prognostic value in ARDS. The advantage of using human plasma is easier accessibility of sampling compared with lung tissue, which is difficult to obtain. The first untargeted proteomics assessment of patients with ARDS was performed in 2004 by Bowler et al. [155] on plasma, edema fluid, and BALF samples collected from patients with ARDS and healthy controls to identify the protein profile. In the acute phase of the disease, several proteins were identified, including albumin, serum amyloid protein, hemopexin, immunoglobulin (Ig)-G heavy chain, complement component 3, α2 or β‑hemoglobin, α2 or β2‑glycoprotein1, and α2‑Heremans‑Schmid‑glycoprotein [155]. Novel biomarkers for ARDS diagnosis/pathophysiology and treatment have been investigated by Chen et al. [151] by dividing the sample into 3 groups: direct lung injury, indirect lung injury, and control. Sixteen proteins were identified; the lung injury groups shared 11 proteins, and 5 proteins were specific to the indirect group. By finding different inflammatory pathways, this study was able to confirm a promising ability of proteomic strategies to provide the pattern of ARDS subphenotypes. In a recent analysis of plasma samples in patients with SARS-CoV-2-induced ARDS, 75% of the 368 proteins measured were significantly upregulated in moderate-severe COVID-19. Of interest, 6 proteins (IL-6, CKAP4, Gal-9, IL-1ra, LILRB4, and PD-L1) were associated with the severity of COVID-19 [162]. Li et al. [153] found 128 upregulated proteins and 34 downregulated proteins in patients with ARDS compared with healthy volunteers, allowing the possible identification of new biomarkers. The association between proteomic analysis and outcome has recently been investigated by Dong et al. [154] who observed that plasma insulin-like growth factor binding protein 7 (IGFBP7) increased ARDS 28-day mortality (odds ratio [OR], 1.11; 95% confidence interval [CI], 1.04–1.19; *p* = 0.002) and that the association between IGFBP7 and ARDS 28-day mortality seems to be mediated by the platelet count (OR, 1.03; 95% CI, 1.02–1.04; *p* = 0.01). Liu et al. [152] suggested that IL-10 can provide prognostic information on outcome in patients with ARDS undergoing ECMO. Other proteomic studies on blood samples in ARDS are reported in Table 3.

#### Bronchoalveolar lavage fluid proteome

The epithelial lining fluid and its proteins cover the airways and alveoli, whereas BALF represents the proteome of airways. Proteomic analysis of BALF revealed that several proteins are modified after lung injury [155]. Regarding the ARDS subphenotypes, a proteomic study investigating BALF in early (< 7 days) and late (> 8 days) survivors and non-survivors after ARDS concluded that a dynamic change of proteins occurred between the early and late timepoints and protein expression differed between survivors and non-survivors [158]. Bowler et al. [155] indicated that albumin, transferrin, IgG, clusterin, serum amyloid protein, α2 and β‐hemoglobin, α2 and β2‐glycoprotein1, α1‐antitrypsin, and α2‐Heremans‐Schmid‐glycoprotein were increased in the BALF of patients with ARDS, whereas SP-A was decreased. Similar protein expression was found by Schnapp et al. [148], including albumin, ceruloplasmin, fibrinogen α, α1 chymotrypsin, α2‐Heremans‐Schmid‐glycoprotein, insulin-like growth factor binding protein-3, and other proteins. Torre et al. [156] confirmed that patients with ARDS express several inflammatory biomarkers in BALF, including apolipoprotein A1 and S100. Chang et al. [149], for the first time, demonstrated a time-dependent modification of proteins during different inflammatory phases of ARDS. Bhargava et al. [158] found that ARDS survivors show a predominance of coagulation and fibrinolysis proteins, immune responsive proteins, and proteins maintaining cation and iron homeostasis. On the other hand, early-phase non-survivors had more proteins of carbohydrate catabolism. Nguyen et al. [157] investigated the BALF proteome of patients with ARDS and ventilator-associated pneumonia (VAP) compared with controls and found that S100A8, lactotransferrin, and actinin 1 are expressed in patients with VAP and ARDS but not in controls and patients without VAP. Yuan et al. [163] found that NADH-ubiquinone oxidoreductase chain 1 (ND-1) was overexpressed in patients with ARDS in comparison with healthy volunteers. Bhargava et al. [160] identified 142 proteins in patients with ARDS, including proteins implicated in injury, repair, and fibrosis such as coagulation/thrombosis, acute phase response, and complement activation, which differed between survivors and non-survivors. Ren et al. [159] found that the protein deleted in malignant brain tumors 1 (DMBT1), a protein implicated in cancer research, can potentially serve as a biomarker for an early ARDS diagnosis and assessment of disease severity. Factor VII activating protease (FSAP) was found to be increased in alveolar macrophages and bronchial epithelial and endothelial cells of lungs of patients with ARDS [164]. In addition, platelet-activating factor is a proinflammatory phospholipid that was found to be increased in the BALF of patients with ARDS, suggesting an alternative route to regulate inflammation [165].

#### Lung tissue proteome

Lung tissue samples are more difficult to obtain than serum and plasma samples, limiting their diagnostic and prognostic value [146]. Proteomics of the lung tissue are still based on in vivo experiments. In a rat ARDS model, overexpression of PRDX1 increased the release of IL-6, IL-8, and TNF-α [166]. Yen et al. [167] showed that, in a rat model, tidal volume was associated with the expression of complement/coagulation cascade proteins, and low end-expiratory lung volumes were associated with expression of mitochondrial respiratory chain protein. They concluded that that tidal stretch and lung collapse can activate different pathways. In a recent large animal study, proteomics of lung tissue revealed differences in inflammation and alveolar‑capillary barrier response between atelectasis and aerated regions. Atelectasis regions showed a predominance of negative enrichment related to the extracellular matrix, immune response, tissue development, stress, and metabolism [168]. In a mice model, Yue et al. [169] observed that the proteomic profile differs between direct lipopolysaccharide-induced lung injury and indirect lung injury. CXCL15 was upregulated in the indirect lung injury group, and liver X receptor/retinoid X receptor activation, nitric oxide expression, and reactive oxygen species in macrophages were activated by the direct injury group. Xu et al. [170] suggested 5152 proteins in lung tissues from oleic acid-treated and saline-treated mice, of which 545 were upregulated and 304 downregulated. Particularly, antithrombin III, 12-lipoxygenase, dedicator of cytokinesis 2, polycystin-2, and plasminogen are new potential biomarkers for ARDS induced by oleic acid. With the advent of the global COVID-19 pandemic, the use of proteomics approaches has made advances, providing further knowledge about the effects of infection. A study on biopsy samples from patients with ARDS induced by COVID-19 revealed that the lung underwent a huge alteration in proteins related to lung inflammation and coagulative dysregulation. In this study, other organs were investigated and showed significant protein alterations [171]. Similarly, Nie et al. [172] found 11,394 proteins in autopsy samples from patients with COVID-19, resulting in overexpression of cathepsin L1 in the lung tissue probably due to hyperinflammation, dysregulation of angiogenesis, coagulation, and fibrosis.

#### Lung cell proteome

As alternative to lung tissue proteome, lung cell proteome (i.e., alveolar macrophages, which represent the main defense of the airway) was collected and analyzed [173]. The role of alveolar macrophages in ARDS has been widely investigated and confirmed, showing that alveolar macrophages probably act as phagocytes for removing the infectious or toxic trigger from the airways [174]. Dong et al. identified 135 proteins, of which 27 were upregulated on alveolar macrophages in the exudative (17 proteins) and recovery (10 proteins) phases of ARDS, potentially serving as biomarkers [161]. No studies investigating alveolar macrophages during each phase of ARDS are currently available.

### Metabolomics

Metabolomics refers to an emerging field targeting the study of a large set of metabolites within a single biological sample in a specific condition. Metabolomics allows detection of physiologic and pathologic changes in the concentration of metabolites using nuclear magnetic resonance spectroscopy, gas chromatography-mass spectrometry, or liquid chromatography-mass spectrometry (LC–MS) or incorporating more than one of these techniques. In contrast to other omics technologies such as proteomics and genomics, fewer metabolites are identified in humans compared with genes or proteins, and thus they are easier to access [175]. In addition, an advantage of metabolomics is that the molecules reflect the upregulation of a specific phase of a biological cascade, allowing eventual pathologic mechanisms to be detected in real time [176]. Metabolomics can be developed for a broad variety of biological samples, including BALF, exhaled breath condensate (EBC), and plasma/serum [21]. Plasma/serum sampling seems to be more suitable for the detection of pathologic metabolites both in pulmonary and extrapulmonary ARDS, whereas BALF can be more specific for identifying the changes in patients with pulmonary ARDS [21]. Problems with metabolomics technology include: (1) high dimensionality, which means that the metabolites are larger than the number of samples, (2) multicollinearity, meaning that metabolites from the same biological sample may be interconnected, (3) variability due to the analytical deviations of the technology that has been used, e.g., LC–MS [177]. Similar to proteomics, metabolomics can be targeted or untargeted. Targeted metabolomics refers to specific metabolites that belong to pathways of interest, and untargeted metabolomics refers to a concomitant measure of several metabolites from biological samples without a specific research question.

#### Exhaled breath condensate

Metabolomics research in ARDS started in 1998 with a study of 19 patients with ARDS and 18 ventilated controls analyzing the EBC to identify that isoprene is an ARDS-associated metabolite [178]. Many years later, Bos et al. [179] identified 3-methylheptane, octane, and acetaldehyde in the EBC of patients with ARDS. Singh et al. [180] found associations with N-acetyl glycoproteins, acetoacetate, lactate, creatinine, histidine, formate, and branched-chain amino acids, and Stringer et al. [181] confirmed the association with ARDS and phosphatidyl serine, total lipids, and total choline. Since the advent of the COVID-19 pandemic, a comparison of metabolomic signatures between patients with H1N1 and patients with COVID-19 with ARDS was performed. It was found that COVID-19 causes a significant deficit in energy supply that activates supplementary energy pathways. On the contrary, patients with H1N1 showed significantly marked inflammatory and oxidative stress responses [182]. A comparison of exhaled breath samples from patients with COVID-19 and non-COVID-19 ARDS revealed that those with COVID-19 present a specific metabolic profile, including volatile compounds methylpent-2-enal, 2,4-octadiene 1-chloroheptane, and nonanal [183].

#### Plasma metabolites

In 2011, Stringer et al. [181] examined the plasma of 13 patients with sepsis-induced lung injury and 6 healthy controls, finding that total glutathione, adenosine, phosphatidylserine, and sphingomyelin are metabolites associated with ARDS induced by sepsis. In 2019, the metabolomic profile of patients with H1N1 influenza was detected, revealing a strict association with the Sequential Organ Failure Assessment (SOFA) and the arterial partial pressure of oxygen/fraction of inspired oxygen (PaO_2_/FiO_2_) of patients with ARDS [184]. The same research group also tested the metabolomic profile in patients with ARDS from other causes [185]. Lin et al. [186] identified 222 metabolites, of which 128 were altered in patients with ARDS in comparison with heathy controls. Phenylalanine, aspartic acid, and carbamic acid levels were significantly different between mild and severe ARDS groups, and ornithine, caprylic acid, azetidine, and iminodiacetic acid may potentially predict the severity of ARDS. Viswan et al. [187] identified biological endotypes of ARDS in 464 patients and controls, and found isoleucine, leucine, valine, lysine/arginine, tyrosine, threonine in BALF, and proline, glutamate, phenylalanine, valine in serum. The association of these biological endotypes with SOFA and APACHE II score produced a robust predictor of mortality for patients with ARDS. Xu et al. [188] investigated the metabolomic profile of 42 patients with ARDS and 28 healthy controls and found an increase in phenylalanine, D-phenylalanine, and phenylacetylglutamine in non-survivors compared with survivors of ARDS.

#### Bronchoalveolar lavage fluid

Rai et al. [189] compared the metabolome of the BALF of 21 patients with ARDS with 9 ventilated patients admitted to the ICU, finding an association with ARDS for BCA, arginine, glycine, aspartic acid, succinate, lactate, glutamate, ethanol, acetate, and proline. Again, on BALF, Evans et al. [190] suggested that guanosine, xanthine, hypoxanthine, lactate, and phosphatidylcholines are associated with ARDS in a comparison with healthy controls. In 2017, Rogers et al. [191] indicated 760 metabolites, of which 235 were significantly higher in patients with ARDS in comparison with those with hydrostatic pulmonary edema. Viswan et al. [192] proposed 6 biomarkers as signatures of ARDS, including proline, lysine/arginine, taurine, and threonine as signs of moderate/severe ARDS, and glutamate as a sign of mild ARDS. In addition, lung metabolism was found to be altered in patients with ARDS with acute kidney injury, suggesting a potential role of peripheral diseases in ARDS metabolic response [193].

### Omics approaches in COVID-19 ARDS research

A multi-omics approach has been speeded up during the COVID-19 pandemic to find alternative treatments. In patients hospitalized with COVID-19, Ang-2, IL6, and MPO were associated with mortality, but without conclusive evidence of specificity for COVID-19. In addition, 207 differentially expressed miRNAs were found between survivors and non-survivors in the severe COVID-19 group, including miRNA pathways for platelet activation, extracellular matrix-receptor interactions, Ras, and ErbB2 [194]. Differently from non-COVID-19 ARDS, patients with COVID-19 showed better outcomes using corticosteroids. This can be explained by the fact that COVID-19 is a highly heterogenous disease with a known cause that may develop into ARDS, thus not so different from classic ARDS [195]. It seems that the combination of biomarkers can characterize the pathophysiologic responses in patients with COVID-19 or individualize management according to the biological phenotypes. Gustafson et al. [194] published a study, providing a clear example of how the incorporation of clinical data with omics should be identifying COVID-19 phenotypes and providing prognostic information. The authors confirmed that corticosteroids are useful in COVID-19 under inflammatory conditions, reinforcing the need for appropriate timing of administration and settings when designing clinical trials. However, as per omics studies, the sample size is limited, and there were several missing data. This highlights even more the need for collaborative networks and biobanks [105].

### Future developments

Omics research seems promising in both preclinical and clinical settings. However, experimental models of ARDS cannot be easily translated into the clinical scenario and should be interpreted with caution. A genetic susceptibility to ARDS and its outcomes has been identified as a potential factor that can interact with the environment, affecting response to treatments, outcomes, and susceptibility to ARDS [196]. In addition, omics approaches are currently unavailable in most laboratories, treatment consequences are poorly known, and the costs are high. On the other hand, omics and biological markers may help better understand the disease, without the need to revise the definition of ARDS. Therefore, identifying subsets with similar biological features and integrating biological traits into ARDS classification may help in finding potential novel therapies [196]. Nevertheless, ARDS research based on omics approaches is still in its infancy. Several factors should be taken into account in implementing and including omics in clinical practice [197]. (1) The role of a collaborative biobank is pivotal. Biobanks of plasma and alveolar samples from patients with ARDS can allow researchers to obtain appropriate samples. To reach this milestone, collaborative networks are urgently needed. (2) Biological samples can be used to test the in vitro efficacy of certain therapies for reverse translational studies. (3) Biological samples should be collected as standard practice in RCTs on patients with ARDS to test and investigate treatments in subphenotypes of patients with ARDS [196]. (4) Biological factors that enrich the population should be measured in interventional studies. (5) The timing of assessment and ARDS evolution should always be recorded when managing biological samples. (6) Post-hoc subphenotypes analysis of RCTs should be implemented to identify biological markers of interest to translate into novel RCTs [196].

## Conclusions

The heterogeneity of ARDS is the main obstacle to finding effective pharmacologic treatments. The identification of ARDS subphenotypes using omic technology offers a new opportunity for the development of diagnostic tools and personalized medicine in ARDS.

## Data Availability

Not applicable.

## Abbreviations

ARDS: Acute respiratory distress syndrome
BALF: Bronchoalveolar lavage fluid
BORCS5: BLOC-1 related complex subunit-5
CI: Confidence interval
DAD: Diffuse alveolar damage
EBC: Exhaled breath condensate
ECMO: Extracorporeal membrane oxygenation
FiO_2_: Fraction of inspired oxygen
GWAS: Genome-wide association study
ICU: Intensive care unit
Ig: Immunoglobulin
IL: Interleukin
LC–MS: Liquid chromatography-mass spectrometry
miRNA: Micro-RNA
mRNA: Messenger RNA
OR: Odds ratio
PaO_2_: Arterial oxygen tension
PEEP: Positive end-expiratory pressure
RCT: Randomized controlled trial
rRNA: Ribosomal RNA
siRNA: Short interfering RNA
SOFA: Sequential Organ Failure Assessment
TNF: Tumor necrosis factor
tRNA: Transfer RNA
VAP: Ventilator-associated pneumonia
